# Does strict invariance matter? Valid group mean comparisons with ordered-categorical items

**DOI:** 10.3758/s13428-023-02247-6

**Published:** 2023-11-29

**Authors:** Winnie Wing-Yee Tse, Mark H. C. Lai, Yichi Zhang

**Affiliations:** Department of Psychology, University of Southern California, 3620 S McClintock Ave, Los Angeles, CA 90089 USA

## Abstract

Measurement invariance (MI) of a psychometric scale is a prerequisite for valid group comparisons of the measured construct. While the invariance of loadings and intercepts (i.e., scalar invariance) supports comparisons of *factor means* and *observed means* with continuous items, a general belief is that the same holds with ordered-categorical (i.e., ordered-polytomous and dichotomous) items. However, as this paper shows, this belief is only partially true—factor mean comparison is permissible in the correctly specified scalar invariance model with ordered-polytomous items but not with dichotomous items. Furthermore, rather than scalar invariance, full strict invariance—invariance of loadings, thresholds, intercepts, and unique factor variances in all items—is needed when comparing observed means with both ordered-polytomous and dichotomous items. In a Monte Carlo simulation study, we found that unique factor noninvariance led to biased estimations and inferences (e.g., with inflated type I error rates of 19.52%) of (a) the observed mean difference for both ordered-polytomous and dichotomous items and (b) the factor mean difference for dichotomous items in the scalar invariance model. We provide a tutorial on invariance testing with ordered-categorical items as well as suggestions on mean comparisons when strict invariance is violated. In general, we recommend testing strict invariance prior to comparing observed means with ordered-categorical items and adjusting for partial invariance to compare factor means if strict invariance fails.

Psychological constructs are unobservable and often indirectly measured by scales with multiple items. For example, the Center for Epidemiologic Studies Depression Scale (*CES-D* Scale; Radloff , 1977) measures the construct of depression using 20 items that assess depressive symptoms (e.g., how often one had a poor appetite during the past week). Social and behavioral researchers commonly use the sum or mean scores of scale items to compare a psychological construct across groups. Using CES-D as an example, past research has compared gender differences in depressive symptoms among adolescents with the sum scores of the scale (Avison & McAlpine, 1992), and examined depression levels among U.S. adults during the COVID-19 pandemic using the mean scores of the scale (Fitzpatrick et al., 2020).

Group comparisons with scale scores are valid only when the observed items measure the same latent construct equivalently across groups, a condition known as *measurement invariance* (MI; Mellenbergh , 1989; Meredith , 1993; Millsap , 2011). MI is an important measurement property that has been widely assessed in psychological and behavioral sciences. In a quick search on PsycINFO, 1,664 peer-reviewed articles published in 2019-2021 contained the keyword “measurement invariance” or “measurement equivalence” in the abstract. If MI does not hold, a condition known as measurement *noninvariance*, differences in scale scores may reflect not only differences in the latent construct of interest but also incomparable measurement across groups, leading to biased estimates of group differences and erroneous inferences. Therefore, MI is a prerequisite for the valid use of scale scores, particularly when evaluating group differences.

Traditionally, popular approaches to MI testing often involve four sequential stages (Widaman & Reise, 1997): *configural* (equality of model structure), *metric* (equality of loadings), *scalar* (equality of loadings and intercepts), and *strict* (equality of loadings, intercepts, and unique factor variances) invariance.^1^ However, in practice researchers often omit applying the test of strict invariance, because scalar invariance supports group comparisons of the *observed means* or *factor means* with continuous items (Meredith & Teresi, 2006; Putnick & Bornstein, 2016; Vandenberg , 2002).^2^ When one or more items are not scalar invariant, group comparisons based on observed means may be biased (Schmitt & Kuljanin, 2008), even though group comparisons of factor means may still be permissible when researchers fit a partial scalar invariance model that correctly adjusts for the noninvariant parameters (Byrne et al., 1989).

As many psychological scale items are not continuous but categorical, researchers have adapted the above multistage procedure to evaluating MI for ordered-categorical items (e.g., Likert-scale questionnaire items; Millsap & Tein, 2004). For example, unlike a continuous measure that can take on an unlimited number of values, a Likert-scale item on “how often one had a poor appetite during the past week” in CES-D often consists of four response categories: *rarely*, *sometimes*, *occasionally*, and *most of the time* (Radloff, 1977). Under the item factor model (Birnbaum , 1968; Wirth & Edwards, 2007), latent responses to ordered-categorical items are continuous but discretized into observed categories by a set of thresholds. As such, modeling ordered-categorical items requires an additional set of threshold parameters, in addition to loadings, intercepts, and unique variances. The intercepts denote the conditional means of the latent response distributions when the latent factor mean is zero and are usually set to zero to define the scale of the latent responses (Wu & Estabrook, 2016), whereas the thresholds indicate the position on the latent trait where a respondent transitions from a lower to a higher category and are often freely estimated (Bovaird & Koziol, 2012). The MI testing procedure for ordered-categorical items parallels the one used for continuous items but with some differences. In particular, while the equality constraints for the configural and metric models are the same, the scalar model often evaluates equality of loadings and *thresholds*, and the strict model tests equality of loadings, thresholds, and unique variances, fixing intercepts at zero in all models (Millsap & Tein, 2004).

With a different distribution, however, ordered-categorical items often involve different MI testing practices than continuous items, including estimation methods (Millsap , 2011; B. O. Muthén , 1984); identification conditions (Millsap & Tein, 2004; Wu & Estabrook, 2016); and parameterization (B. O. Muthén , 2002). In addition, dichotomous items (i.e., with two categories) have different properties and, therefore, involve different practices than ordered-polytomous items (i.e., with three or more ordered categories). An example of this difference is that dichotomous items require additional constraints for identification than ordered-polytomous items (Millsap & Tein, 2004; Wu & Estabrook, 2016).

## Does scalar invariance support mean comparisons with ordered-categorical items?

While scalar invariance allows factor mean and observed mean comparisons with continuous items, the question remains as to whether or not the same practice generalizes to both dichotomous and ordered-polytomous items. Many methodological guidelines have suggested that scalar invariance supports factor mean comparisons with ordered-categorical items (e.g., Bauer , 2017; Bovaird & Koziol, 2012; Bowen & Masa, 2015; Kite et al. , 2018; Putnick & Bornstein, 2016), and some studies have further advised that scalar invariance allows observed mean comparisons with such items (e.g., Svetina et al. , 2019). A general belief is that “scalar invariance supports cross-group comparisons of manifest (or latent) variable means on the latent variable of interest” (Svetina et al. , 2019, p. 2). As such, strict invariance, the most stringent invariance condition, is often considered “optional” (Pendergast et al. , 2017, p. 71) and is “rarely pursued” (Svetina et al. , 2019, p. 2).

For these reasons, tutorials on MI testing with ordered-categorical items often include only tests of configural, metric, and scalar invariance, but not strict invariance (e.g., Bowen & Masa, 2015; Pendergast et al. , 2017; Svetina et al. , 2019). Moreover, in the popular software Mplus for latent variable modeling, the convenient MODEL option for MI testing supports only up to scalar invariance for both dichotomous and ordered-polytomous items (L. K. Muthén & Muthén, 1998–2017, 2013). Such an option may encourage users to stop invariance testing at the scalar invariance stage for ordered-categorical items; however, researchers can still manually define a strict invariance model in Mplus.

Whereas the common presumption is that scalar invariance supports both factor and observed mean comparisons with ordered-categorical items, opposing arguments have maintained that strict invariance is required for some forms of mean comparisons. Liu et al. (2017) proved that strict invariance is necessary to ensure that differences in the *observed means* of ordered-categorical items are attributable to only the differences in the latent construct. In other words, valid comparisons of observed means require invariance of loadings, thresholds, intercepts, and unique variances for both dichotomous and ordered-polytomous items. On the other hand, Wu and Estabrook (2016) noted that scalar invariance supports *factor mean* comparisons specifically for ordered-polytomous items, although they did not discuss the dichotomous case. For dichotomous items, however, little is known in the literature on whether factor mean comparisons are valid in the scalar invariance model.

Given inconsistent guidelines and limited research on the invariance condition required for observed and factor mean comparisons with ordered-categorical items (Pendergast et al., 2017), there is a need to bring clarity to the question of whether strict invariance is a prerequisite for factor mean and observed mean comparisons. Moreover, dichotomous and ordered-polytomous items are often considered together, implicitly or explicitly, in a broader type of “ordered-categorical” items. However, whether the same practices apply to both types of items also remains a question.

### The current study

To fill that gap in the literature, the current paper discusses and evaluates the necessary MI condition for valid observed and factor mean comparisons with dichotomous and ordered-polytomous items. As illustrated, unlike the cases for continuous items, strict invariance is necessary when the goal is to compare observed means of both dichotomous and ordered-polytomous items. Moreover, factor mean comparisons are valid in the scalar or partial scalar model with ordered-polytomous items but not dichotomous items; for the latter, the strict or partial strict model is needed for valid factor mean comparison, as demonstrated in the simulation results.

We begin with a brief review of MI testing practices as reported in the literature and present an illustrative example showing that observed mean and factor mean comparisons can provide diverging results. We then define the stages of invariance testing for ordered-categorical items. Next, we perform a Monte Carlo simulation study to systematically evaluate the impact of strict noninvariance on observed and factor mean comparisons. Even when all items are strict invariant, using only a scalar invariance model can introduce bias in the estimation of factor mean differences. Lastly, we provide a tutorial on MI testing with ordered-categorical items, including a demonstration of how to establish partial invariance when needed and how to perform factor mean comparisons when strict invariance fails.

## Strict invariance was not commonly tested in the literature

We performed a brief review of MI testing practices with ordered-categorical items in the psychological-related research, with a focus on studies that evaluated MI using multigroup confirmatory factor analysis (MG-CFA) with weighted least squares (WLS).^3^ From a search on the PsychINFO database using the following keywords: (“measurement invarian*” OR “factorial invarian*” OR “differential item function*”) AND (WLS* OR “diagonally weighted” OR DWLS OR Categorical OR Ordinal OR binary OR Likert), we identified 74 peer-reviewed articles published in 2017 and 2018. Fifteen of them were excluded because they (a) were not written in English ($n$ = 3), (b) did not test MI using empirical data ($n$ = 10), (c) did not treat scale items as ordered-categorical ($n$ = 1), or (d) were a corrigendum of a previously published article ($n$ = 1). Thirty-one of the remaining articles tested MI using MG-CFA, and the rest of them evaluated MI within the item response theory (IRT) framework or used other approaches (i.e., bootstrap, moderated nonlinear factor analysis, or multiple indicator multiple cause modeling).

Among the 31 articles that used MG-CFA, three involved dichotomous items, and 28 included ordered-polytomous items with more than three response categories. These articles used either a variant of the diagonally weighted least square estimation (DWLS; $n = 24$) or a variant of the maximum likelihood estimation (ML; $n = 3$),^4^ but four of them did not specify the estimation method. Whereas some (41.94%) of the articles evaluated strict invariance, the majority (58.06%) of them tested up to the model of configural ($n = 1$), metric ($n = 2$), or scalar invariance ($n = 15$). Finally, a handful of the articles further compared observed means ($n = 7$) or factor means ($n = 10$) across groups. Two of these articles compared observed means of the ordered-polytomous items without establishing strict invariance, and one compared factor means of dichotomous items in the scalar invariance model.

This brief review shows that the test of strict invariance was often missed when testing MI for scales with ordered-categorical items. In addition, we found instances of observed mean comparisons with ordered-categorical items without the support of strict invariance and an instance of factor mean comparison with dichotomous items in the scalar invariance model.

**Table 1:** Observed and factor mean comparison in the illustrative example

	Mean difference	Model	Estimate [$95\%$ C.I.]
	Observed		-0.02 [-0.01, 0.06]
Dichotomous	Factor	Scalar	0.26 [-0.54, 1.12]
		Partial strict	**0.33 [0.04, 0.76]**
	Observed		-0.07 [-0.04, 0.19]
Ordered-polytomous	Factor	Scalar	**0.15 [0.02, 0.3]**
		Partial strict	**0.14 [0.02, 0.3]**

*Note.* Bolded figures are the statistically significant estimated mean difference

## An illustrative example

The following example shows how inferences of comparing observed means and factor means can diverge due to noninvariance in unique variances. To illustrate, we simulated data based on an empirical study by Sharman et al. (2019), who developed The Beliefs About Crying Scale (BACS), a psychological scale that measures beliefs about whether crying is a helpful or unhelpful behavior in individual and social contexts. For simplicity, we focus here on the one-dimensional Helpful subscale of BACS and compare means between males and females to examine the role of unique factor invariance in mean comparisons.

The Helpful subscale has seven ordered-polytomous items, each with five response categories (1–5). To create an example for dichotomous items, the response categories below 3 were collapsed into 0, and those at or above 3 were collapsed into 1. As will be illustrated in the tutorial of this paper, the Helpful subscale achieves partial strict invariance with ordered-polytomous items and achieves strict invariance with dichotomous items.

We used the parameter estimates from Sharman et al. (2019) to simulate two toy datasets, one for dichotomous items and another for ordered-polytomous items with five categories. We simulated the datasets to have invariant loadings and thresholds but noninvariant unique variances in the last three items between the two groups. In other words, the simulated datasets achieve scalar invariance but not strict invariance. Each dataset had a sample size of 1000, and the goal was to detect an assumed population mean difference of 0.2. The full R script for the simulation is available in the supplemental materials.

We evaluated the observed mean difference by performing an independent sample *t* test on the mean scores of the seven items between males and females. Furthermore, we examined the factor mean difference estimate, $\hat{\alpha }_f$, in two models: (a) the scalar model, which allows the unique variances to freely vary, and (b) the partial strict model, which constrains the unique variances to be equal except for the noninvariant items. To allow for factor mean comparison, we fixed the factor mean of the male group at 0; thus, the factor mean of the female group indicated the difference between the two groups. Note that neither the *t* test nor the scalar invariance model accounted for the noninvariance of unique variances, whereas the partial strict model did.

As shown in Table 1, the result of the observed mean comparison did not agree with that of the factor mean comparison. For dichotomous items, the independent sample *t* test failed to detect a difference in observed means between the two groups, *t*(998) = 1.40, *p* = 0.16; similarly, the Wald test in the scalar model also failed to detect a factor mean difference between the two groups, *z* = 0.69, *p* = 0.49. However, the Wald test in the partial strict model detected a factor mean difference between the two groups, *z* = 2.19, *p* < .05. For ordered-polytomous items, whereas the independent sample *t* test failed to detect an observed mean difference, *t*(998) = 1.23, *p* = 0.22, the Wald test in both the scalar, *z* = 2.28, *p* <.05, and partial strict models, *z* = 2.20, *p* <.05, detected a factor mean difference.

The above example illustrates a case where the conclusions of mean comparisons diverged in different models even when the data was scalar invariant. While in practice the population mean difference is unknown, the question lies in which conclusion is valid if some items have noninvariant unique variances. In the following, we will review MI testing with ordered-categorical items and systematically evaluate the impact of unique factor noninvariance on mean comparisons with a simulation study.

## Measurement invariance testing

MI testing typically involves a multistage procedure that sequentially evaluates nested models each of which has additional equality constraints across groups. This procedure was originally developed for continuous items under the multivariate normality assumption within a common-factor model (Horn & McArdle, 1992; Meredith , 1993; Vandenberg , 2002; Widaman & Reise, 1997). Since ordered-categorical items do not fulfill such distributional assumptions, alternative multistage procedures were established within the item factor model framework (Liu et al. , 2017; Millsap & Tein, 2004; Svetina et al. , 2019; Wirth & Edwards, 2007). In this section, we begin by defining the common factor model and the item factor model and then discuss the MI testing procedures for ordered-categorical items.

### Common factor model with continuous items

Let $Y_{ij}$ ($i$ = 1, 2, $\ldots$, $N$; $j$ = 1, 2, $\ldots$, $p$) be the response of the $i$th person on the $j$th item in a scale of $p$ items measuring a latent common factor $\eta$. A measurement model links $Y$ and $\eta$ probabilistically with a set of parameters, expressed as $P(Y_{ij} | \eta )$. Formally, MI holds when the conditional distribution of the observed items is the same across subgroups, such as gender and ethnicity (Mellenbergh , 1989; Meredith , 1993). That is, for a subgroup membership variable $G$,

1 $$\begin{aligned} P(Y_{ij} | \eta _i, G_i = g) = P(Y_{ij} | \eta _i), \forall j, g. \end{aligned}$$

In other words, responses to scale items depend solely on the common factor but not the group membership. For example, two people with the same beliefs about crying should have the same propensity to respond to the scale items similarly, regardless of their group membership.

For continuous items, the common factor model (Thurstone , 1947) is usually used, represented as

2 $$\begin{aligned} Y_{ij} = \nu _j + \lambda _j \eta _{i} + \varepsilon _{ij}, \end{aligned}$$

where $\nu _j$ is the measurement intercept, $\lambda _j$ is the factor loading, and $\varepsilon _{ij}$ is the realized value of the unique factor. It is commonly assumed that $\varepsilon _j$ is normally distributed with constant variance $\theta _{j}$, so that $Y_{ij}$ is also normally distributed conditioned on $\eta _i$. In addition, the local independence assumption is usually applied such that, when conditioned on $\eta _i$, ${{\,\mathrm{\textrm{Cov}}\,}}(Y_{ij}, Y_{ij'} | \eta _i) = 0$ for $j \ne j'$. When there are $K$ groups, the model is

3 $$\begin{aligned} Y_{ijk} = \nu _{jk} + \lambda _{jk} \eta _{ik} + \varepsilon _{ijk}, \end{aligned}$$

where $$k$$ = 1, 2, $$\ldots $$, $$K$$, and $${{\,\mathrm{\textrm{Var}}\,}}(\varepsilon _{ijk})$$ = $$\theta _{jk}$$.

When a common factor model holds, MI requires that the measurement parameters, for example $\nu _j$, $\lambda _j$ and $\theta _{j}$ for the model in Eq. 2, are the same across groups (e.g., Meredith, 1993). For continuous variables, valid group comparisons do not require all measurement parameters to be equal across groups. Conventionally, researchers have distinguished between four stages of measurement invariance: (a) configural invariance, which requires the configuration of the factor loadings to be the same across groups (Horn & McArdle, 1992; b) metric/weak invariance, which requires equal factor loadings (i.e., $\lambda _{jk}$ = $\lambda _j$ for all $j$s and $k$s) in addition to configural invariance; (c) scalar/strong invariance, which requires equal measurement intercepts (i.e., $\nu _{jk}$ = $\nu _j$ for all $j$s and $k$s) in addition to metric invariance; and (d) strict invariance, or strict factorial invariance, which requires all measurement parameters ($\nu _j$, $\lambda _j$, and $\theta _j$ for all $j$s) to be equal across groups.

### Item factor model with ordered-categorical items

**Fig. 1:**
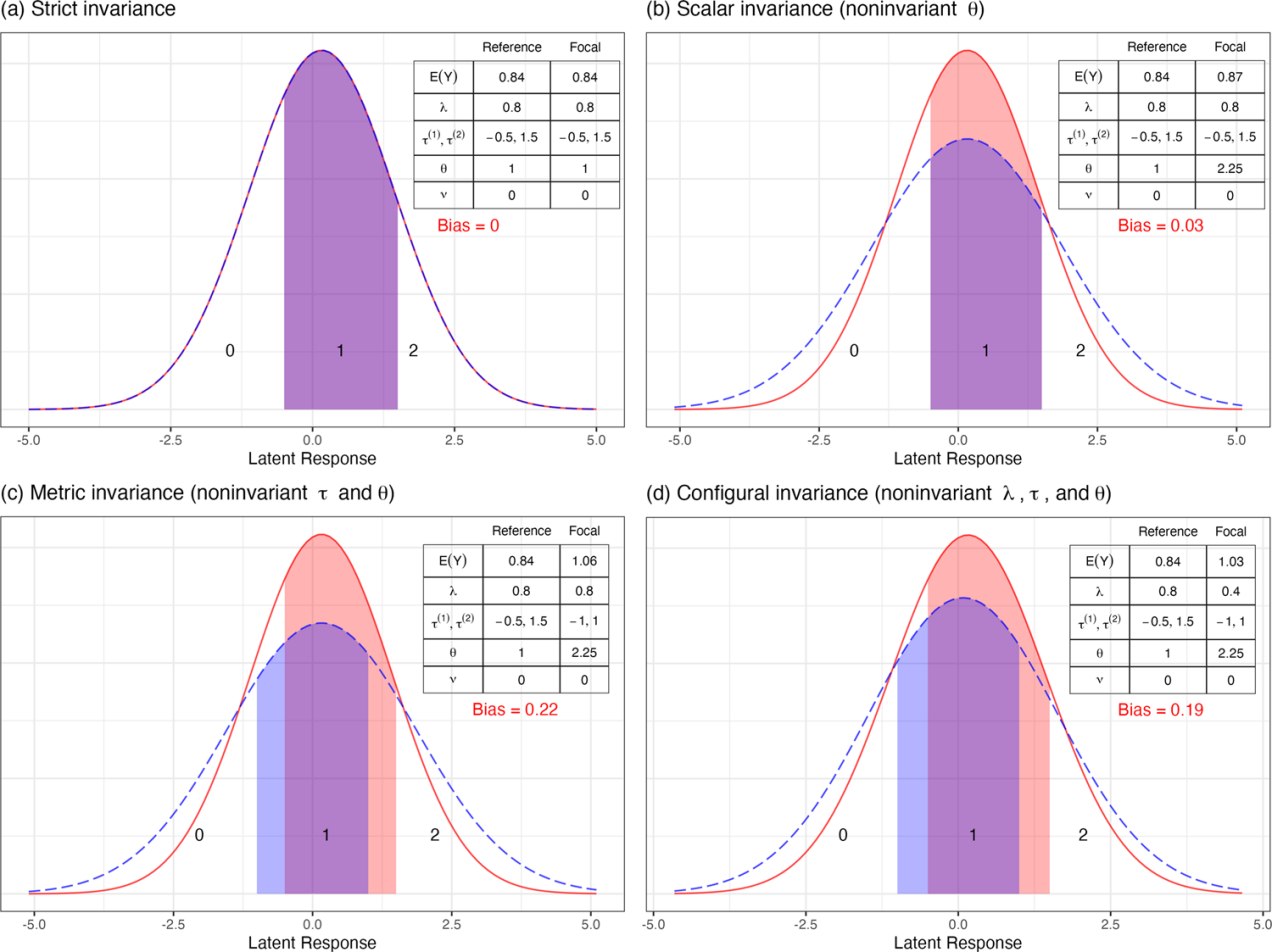
An illustration of biases due to noninvariance in different parameters. All plots show the latent response distributions of an item with three response categories, scoring 0, 1, and 2. Both the reference group (*red*, *solid line*) and the focal group (*blue*, *dashed line*) share the same factor mean ($\alpha = 0.2$) and factor variance ($\psi = 1$). Regions below the first threshold, between the first and second thresholds, and above the second threshold indicate the probability of scoring 0, 1, and 2, respectively. E(Y) = observed mean. $\lambda$ = factor loading. $\tau ^{(1)}, \tau ^{(2)}$ denote the first and second thresholds. $\theta$ = unique factor variance. $\nu$ = intercept. Bias = observed mean difference due to noninvariance in different parameters while the population mean difference is 0

Let $y_{ij}$ be the observed categorical response and $y^*_{ij}$ be the latent continuous response of the $i$th person for item $j$, under the item factor model:

4 $$\begin{aligned} Y^*_{ij} = \nu _j + \lambda _j \eta _i + \varepsilon _{ij}, \end{aligned}$$

where $\eta _i$ is the latent common factor, $\nu _j$ is the latent intercept, $\lambda _j$ is the factor loading, and $\varepsilon _{ij}$ is the realized value of the unique factor. The equation is the same as the factor model for continuous variables. From here, however, it is assumed that $Y^*_{ij}$ is mapped to $Y_{ij}$, the observed variable with $C - 1$ thresholds and $C$ categories (0, 1, $\ldots$, $C - 1$), by a cumulative link function such that

5 $$\begin{aligned} Y_{ij} = {\left\{ \begin{array}{ll} 0 &{} \text{ if } Y^*_{ij} \le \tau _{j}^{(1)} \\ c &{} \text{ if } \tau _{j}^{(c)} < Y^*_{ij} \le \tau _{j}^{(c + 1)} \\ C-1 &{} \text{ if } Y^*_{ij} > \tau _{j}^{(C-1)} \end{array}\right. }, \end{aligned}$$

where $\tau _{j}^{(1)}$, $\ldots$, $\tau _{j}^{(C-1)}$ are the threshold parameters for the $j$th item. For example, consider that the latent responses, $Y^*$, to the item “crying makes me feel better” take a normal distribution. As shown in Fig. 1, the latent responses under $\tau ^{(1)}$ fall in Category 0 (e.g., “Not true for me at all”), those between $\tau ^{(1)}$ and $\tau ^{(2)}$ are in Category 1 (e.g., “Moderately”), and those above $\tau ^{(2)}$ are in Category 2 (e.g., “Extremely true for me”). The item factor analysis model assumes that an observed response is “Extremely true for me” if the latent response lies above $\tau ^{(2)}$.

With a probit link,^5^ it is assumed that $\varepsilon$ follows a normal distribution,

6 $$\begin{aligned} \varepsilon _{ij} \sim \mathcal {N}(0, \theta _j). \end{aligned}$$

which implies that $Y^*_{ij}$, conditioned on $\eta _i$, is normally distributed:

7 $$\begin{aligned} y^*_{ij} | \eta _i \sim \mathcal {N}(\nu _j + \lambda _j \eta _i, \theta _j). \end{aligned}$$

### Measurement invariance testing with ordered-categorical items

Millsap and Tein (2004) identified four types of parameters, $\nu _j$, $\tau _j$, $\lambda _j$, and $\theta _j$, for MI testing with ordered-categorical items. As for continuous items, methodologists (Liu et al., 2017; Millsap, 2011; Millsap & Tein, 2004; Svetina et al., 2019) have proposed similar multistage procedures for ordered-categorical items. These procedures also compare nested models by adding equality constraints of parameters, but they differ in the identification conditions, parameterizations, and order of tests of invariance.

Unlike the procedure used with continuous items, MI testing with ordered-categorical items involves, additionally, $\tau _j$. A typical option to identify an item factor model is by setting $\nu _j$ to zero (Liu et al. , 2017; Millsap & Tein, 2004), which is the default of popular statistical programs Mplus and *lavaan* in R. Alternative identification conditions allow estimations of $\nu _j$ (e.g., Svetina et al. , 2019). Interested readers are referred to Wu and Estabrook (2016) for a comprehensive discussion on identification conditions of item factor models with constraints on different types of parameters. Furthermore, B. O. Muthén (2002) discussed two parameterizations for defining the scales of ordered-categorical items: delta and theta. To allow the test of strict invariance, Millsap and Tein (2004) recommended theta parameterization, with which unique variances are estimable parameters.

Millsap and Tein (2004) introduced a procedure that evaluates invariance models in the following order: (a) configural invariance, (b) invariance of loadings (metric/weak), (c) invariance of loadings and thresholds (scalar/strong) and (d) invariance of loadings, thresholds, and unique variances (strict). This order of invariance tests is also popular in literature (e.g., Liu et al. , 2017; B. O. Muthén , 2002; Pendergast et al. , 2017), although the test of strict invariance is often considered optional (Bowen & Masa, 2015; Pendergast et al. , 2017; Svetina et al. , 2019). In an alternative order of tests, the test of threshold invariance comes before the test of loading invariance (Svetina et al. , 2019; Wu & Estabrook, 2016). Moreover, for dichotomous items, because the metric model is an equivalent model to the configural model, the invariance of loadings and thresholds are usually tested together (Millsap & Tein, 2004; Wu & Estabrook, 2016), resulting in only three stages: configural, scalar, and strict (B. O. Muthén , 2002; Putnick & Bornstein, 2016).

### Observed mean comparison

Just as with continuous items, configural invariance and metric invariance do not support observed mean comparisons with ordered-categorical items. As shown in Fig. 1d, even with the same common factor mean $\alpha = 0.2$, the differences in loadings, thresholds, and unique variances yield different observed scores of an ordered-categorical item in the two groups. Such differences are not attributable to the group difference in the latent construct, but merely due to measurement artifacts when the ordered-categorical item is measured differently between groups. Similarly, Fig. 1c shows that when the thresholds are unequal, the two groups can have different observed scores. If two persons have a latent response at around 1.25, the person from the reference group (red, solid line) would endorse Category 1, as 1.25 falls below $\tau ^{(2)}_r = 1.5$, but the person from the focal group (blue, dashed line) would choose Category 2, as 1.25 falls above $\tau ^{(2)}_f = 1$. Hence, threshold noninvariance results in different probabilities of item endorsement as well as observed scores of an ordered-categorical item.

Although for continuous items scalar invariance supports observed mean comparisons, strict invariance is required for ordered-categorical items. Even when both loadings and thresholds are invariant across groups, the differences in observed responses of ordered-categorical items do not necessarily reflect the differences in the latent responses or the latent common factor (Liu et al. , 2017). As shown in Fig. 1b, due to unequal unique variances, the probability of choosing any of the three response categories differs. This can result in a difference in observed scores, even if the two groups share the same factor mean. When strict invariance holds, differences in the observed means are entirely attributable to the differences in the latent common factor (Liu et al. , 2017). Figure 1a shows that the latent distributions of the two groups align when strict invariance holds. Only in this situation do the probabilities of endorsing each response category overlap between groups, hence accurately reflecting the fact that the two groups share the same standing in the latent construct. The unique variance parameter generally affects the distribution and hence the expected value of the observed responses, except when the distributions are symmetric for all groups. In Appendix A, we present and discuss the mathematical details that support these conclusions.

To summarize, observed mean comparisons with ordered-categorical items require full invariance in loadings, thresholds, intercepts, and unique variances to accurately infer differences in the latent common factor. If any of the items are not strict invariant (i.e., partial strict invariance) distributed, observed means can be different across groups even if they share the same common factor mean. Without full strict invariance, one should consider comparing the factor means.

### Factor mean comparison

To allow factor mean comparisons, it is important to first ensure that the identification condition does not involve fixing all factor means to be zero across groups (Wu and Estabrook , 2016). One way to identify the model is by fixing the factor mean of one group (i.e., reference group) to zero and freely estimating the factor mean of the other groups (i.e., focal groups). The estimated factor mean of a focal group reflects the factor mean difference between the focal group and the reference group.

Factor mean comparisons are permissible in the scalar or partial scalar invariance model for ordered-polytomous items, but only in the strict or partial strict invariance model for dichotomous items. For ordered-polytomous items, as scalar invariance equates the scales of the latent responses, group differences in the factor means reflect group differences in the latent common factor (Wu & Estabrook, 2016). If some thresholds are invariant but some are not, factor means can be compared in the partial scalar invariance model that correctly frees the noninvariant thresholds and constrains the invariant thresholds to be equal across groups.

**Table 2:** Practices for valid mean comparisons

	Observed mean comparison is valid	Factor mean comparison is valid
Ordered-polytomous		In the correctly specified scalar or partial scalar invariance model
Dichotomous	When the data establish strict invariance	In the correctly specified strict or partial strict invariance model

For dichotomous items, however, using the scalar or partial scalar invariance model does not ensure valid factor mean comparisons. When unique variances are allowed to freely vary across groups, the scalar or partial scalar invariance model fails to uniquely identify factor means of the focal groups, even if the model correctly constrains invariant loadings, intercepts, and thresholds to be equal across groups. Contrarily, with additional equality constraints on unique variances, the strict or partial strict invariance model uniquely identifies factor means of the focal groups. Therefore, valid factor mean comparisons require correct equality constraints on the invariant unique variances in addition to loadings, intercepts, and thresholds. Appendix B shows the mathematical support for factor mean comparisons with ordered-polytomous and dichotomous items.

Table 2 summarizes the practices required for valid mean comparisons with ordered-polytomous and dichotomous items. If the goal is to compare observed means, the data must establish strict invariance for both ordered-polytomous and dichotomous items. Whereas factor mean comparisons with ordered-polytomous items are permissible in the scalar or partial scalar invariance model, such comparisons with dichotomous items are valid only in the strict or partial strict invariance model.

**Table 3:** Parameter values for data generation

C	Item(s)	$\lambda$	$\theta _r$	$\tau$	Proportion ($\%$)	Skewness
**Negatively skewed**						
2	1-10	0.6	0.64	-0.59	(28, 72)	-1.00
5	1-10	0.6	0.64	-1.55, -1.08, -0.55, 0.15	(6, 8, 15, 27, 44)	-1.00
7	1-10	0.6	0.64	-1.65, -1.23, -0.92, -0.61, -0.28, 0.3	(5, 6, 7, 9, 12, 23, 36)	-1.00
**Positively skewed**						
2	1-10	0.6	0.64	0.59	(72, 28)	1.00
5	1-10	0.6	0.64	-0.151, 0.553, 1.08, 1.555	(44, 27, 15, 8, 6)	1.00
7	1-10	0.6	0.64	-0.305, 0.279, 0.613, 0.915, 1.227, 1.645	(36, 23, 12, 9, 7, 6, 5)	1.00
**BACS**						
	1	2.68	1	-2.92	(0.2, 99.8)	-1.92
	2	2.16	1	-2.66	(0.4, 99.6)	-2.18
	3	2.21	1	-2.13	(1.7, 98.3)	-1.58
	4	1.83	1	-2.01	(1.8, 98.2)	-1.89
2	5	1.43	1	-2.37	(0.9, 99.1)	-2.93
	6	1.38	1	-1.41	(7.9, 92.1)	-1.46
	7	1.31	1	-1.41	(7.9, 92.1)	-1.52
	1	2.68	1	-3.34, -1.84, -0.22, 2.27	(0, 3.2, 38.2, 57.4, 1.2)	-0.53
	2	2.16	1	-3.29, -1.99, -0.69, 0.99	(0.1, 2.3, 22.1, 59.4, 16.2)	-0.68
	3	2.21	1	-3.58, -1.90, -0.33, 1.32	(0, 2.8, 34.1, 53.7, 9.3)	-0.46
	4	1.83	1	-3.31, -1.94, -0.41, 1.37	(0, 2.6, 31.6, 57.3, 8.5)	-0.53
5	5	1.43	1	-3.47, -1.88, -0.74, 0.57	(0, 3, 19.9, 48.8, 28.3)	-0.64
	6	1.38	1	-2.09, -1.15, -0.34, 1.25	(1.8, 10.8, 24.1, 52.7, 10.6)	-0.58
	7	1.31	1	-2.36, -1.10, -0.53, 0.77	(0.9, 12.6, 16.2, 48.4, 21.9)	-0.62

*Note*. Parameter values used to generate data with negatively skewed distributions, positively skewed distributions, and based on the BACS example. *C* = number of response categories. $\lambda$ = loadings. $\theta _r$ = unique variances for the reference group. Unique variances for the focal groups may change depending on the simulation conditions. $\tau$ = thresholds. Proportion ($\%$) = proportions of endorsing response categories of 0 and 1 for $C = 2$, 0 to 4 for $C = 5$, and 0 to 6 for $C = 7$

## Simulation study

We conducted a Monte Carlo simulation study to evaluate the observed and factor mean differences when scale items demonstrate unique factor noninvariance. The goal was to address the following two main questions: (a) How does the lack of strict invariance impact the statistical inference and estimation of the observed mean difference between groups with ordered-categorical items? and (b) Does the scalar model give an accurate estimate and inference of the factor mean difference for both dichotomous and ordered-polytomous items?

In this simulation study, we examined the impact of unique factor noninvariance on items with two, five, or seven response categories, which are common item types in psychological scales. We used three sets of parameter values to generate observed data (a) with negatively skewed distributions, (b) with positively skewed distributions, and (c) based on an empirical example. Table 3 summarizes the parameter values for data generation, as well as the skewness of the observed response distribution and the proportion of endorsing each response category. For (a) and (b), we adapted parameter values in the Sass et al. (2014) to simulate data of ten items. For ease of comparison, we maintained a constant skewness in the observed response distribution across item types. For (c), as a follow-up of the Illustrative Example, we simulated data of seven items based on the parameter estimates from the Helpful subscale of BACS to systematically evaluate the impact of unique factor noninvariance on empirical data. As most BACS items have a negatively skewed distribution, the result patterns for the simulated BACS data are expected to be similar to those for negatively skewed data.

To isolate the effect of unique factor noninvariance, we simulated data that are scalar invariant but noninvariant in unique variances between groups. Specifically, the focal group had a larger unique variance than the reference group. We defined the mean difference as the mean of the focal group minus the mean of the reference group. Based on these definitions and the analytic results discussed above, we expect the following:

1. In the conditions with unique factor noninvariance, observed mean difference will be underestimated for the simulated data with negatively skewed distributions and overestimated for the simulated data with positively skewed distributions.
2. For dichotomous items, as the scalar invariance model is unidentified, the factor mean estimate will be biased in this model, whether or not the data achieve strict invariance. However, using the correctly specified strict (or partial strict) invariance model will give an unbiased estimate of the factor mean difference.
3. For ordered-polytomous items (items with five or seven categories), using either the correctly specified scalar or strict (or partial strict) invariance model will produce unbiased estimates of factor mean differences.

### Simulation design factors

We manipulated five design factors: group size, number of noninvariant items ($p_{ni}$), degree of noninvariance ($d_{ni}$), population factor mean difference ($\alpha _f$), and number of response categories (C). Similar to previous simulation studies (Hsiao & Lai, 2018; Yoon & Lai, 2018), we set the group size ($n_k$) to 100, 200, and 500. With two groups, therefore, the total sample sizes were 200, 400, and 1000, indicating relatively small, medium, and large sample sizes, respectively.

With reference to the simulation design in Liu and West (2018), we simulated data to have zero, one, and three items that demonstrated unique factor noninvariance in the same direction. The numbers of noninvariant items ($p_{ni}$) corresponded to 0, 10, and 30% of the ten items (first and second sets of parameter values) and 0, 14, and 43% of the seven items (third set of parameter values) with larger unique variances in the focal group than in the reference group, reflecting an absence, a small amount, and a large amount of noninvariance, respectively. Similar to the design in Liu and West (2018), in the conditions with noninvariant items, the focal group had $1.25^2$ or $1.5^2$ times larger unique varaince(s) than the reference group, indicating a small or a large degree of noninvariance ($d_{ni}$).

Following conventional practices with model identification, we fixed the factor mean of the reference group at 0. As such, the factor mean difference between the two groups was equivalent to the factor mean of the focal group ($\alpha _{f}$). The population factor mean of the focal group was set at 0, 0.2, and 0.5, similar to the design in Lai et al. (2021). We simulated items that have two, five, and seven response categories ($C$ = 2, 5, 7).

### Data generation

We used the *SimDesign* package (Chalmers & Adkins, 2020) in R (R Core Team, 2022; version 4.1.3) to structure the simulation. For each design condition, we generated 2500 data sets for analysis.

Assuming a single underlying factor, we simulated the latent common factors ($\eta _{ijk}$) from a normal distribution with a variance of one for both groups. The common factor mean was set at 0 for the reference group ($\alpha _r = 0$) and varied depending on the design conditions for the focal group ($\alpha _f$). The continuous latent responses for each item were generated based on Eq. 4, where both groups shared the same intercepts of 0 ($\nu _{jr} = \nu _{jf} = 0$) and the same loadings ($\lambda _{jr} = \lambda _{jf}$). The unique factors ($e_{jk}$) were simulated from a normal distribution with a mean of zero for both groups. For the reference group, the unique factor variances were 1 ($\theta _{jr} = 1$); for the focal group, the unique factor variances ($\theta _{jf}$) varied according to the design conditions. Lastly, we used the same set of thresholds ($\tau ^{(c)}_{jr} = \tau ^{(c)}_{jf}$) for both groups to convert the latent responses into observed responses with two, five, or seven categories based on Eq. 5.

### Data analysis

Per generated data set, we compared the observed means and factor means between groups. For observed mean comparison, we computed the average item score across the seven items per individual, $\bar{Y}_{ik} = \frac{1}{P}\sum _{j = 1}^P Y_{ijk}$ for $P$ items, and the observed means across individuals in each group, $\bar{Y}_k = \frac{1}{n_k}\sum _{i=1}^{n_k} \bar{Y}_{ik}$.^6^ We then performed an independent sample *t* test in R to test against the null hypothesis that the population observed mean difference was zero at $\alpha = .05$. For factor mean comparisons, we used *lavaan* (Rosseel, 2012) to analyze the data with (a) a correctly specified scalar invariance model and (b) either a correctly specified strict or partial strict invariance model, depending on the design conditions. All models were identified with the default identification conditions in *lavaan* and the theta parameterization to allow for free estimation of unique variances.^7^ In all models, we examined the factor mean estimate and statistical significance of the focal group, which denoted the factor mean difference as the factor mean of the reference group was set at 0.

**Fig. 2:**
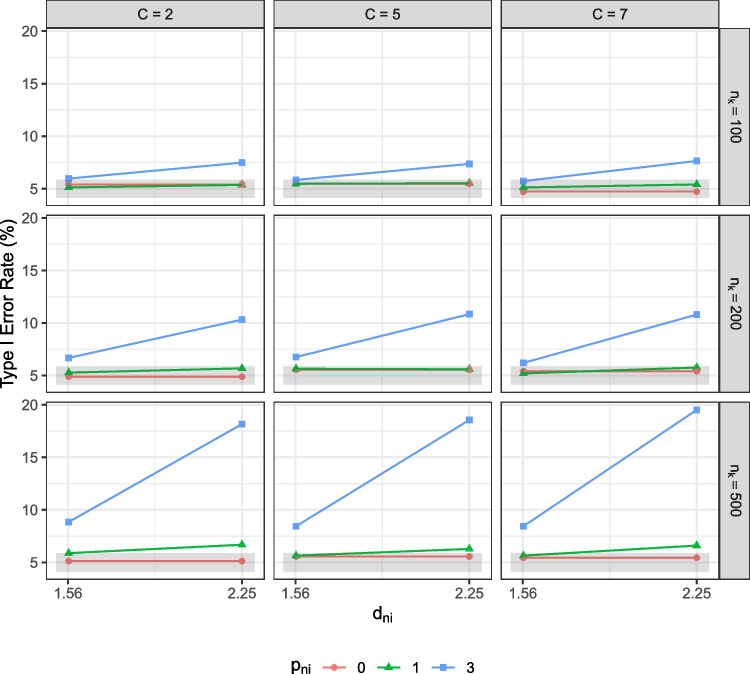
Type I error rate of the observed mean comparisons. $n_k$ = group size. *C* = number of response categories. $p_{ni}$ = number of unique factor noninvariant items. $d_{ni}$ = degree of unique factor noninvariance. $\alpha _{f}$ = population factor mean of the focal group. The *shaded area* is the acceptable range of type I error rates, 4.13–5.87%, in this study

We summarized the simulations in terms of rejection rate and raw bias of the observed or factor mean differences. Rejection rate denotes the type I error rate or power when the population factor mean difference was zero or nonzero, respectively. The expected standard error of the current simulation was .44%, calculated using $\sqrt{(1 - \alpha )\alpha / R}$ (Sass et al., 2014) with $R = 2,500$ and $\alpha = 5\%$. Therefore, we determined that the acceptable range for type I error rates was 4.13%-5.87%—two standard errors away from the nominal 5% alpha level. As power is a function of sample size, we compared other conditions with noninvariant items against the baseline conditions that had zero noninvariant items to evaluate the impact of unique factor noninvariance. The raw bias was the average deviation of the sample observed or factor mean difference from the population mean difference across the replications.

### Simulation results

The result patterns for the simulated BACS data, of which most items have a negatively skewed distribution, were the same as those for the negatively skewed data. The result patterns between negatively skewed and positively skewed data were highly similar, except that the directions of biases in the observed mean difference varied. As expected, although the magnitudes of biases were similar, the observed mean difference was underestimated for negatively skewed data and overestimated for the positively skewed data. For observed mean comparison, the result patterns of type I error rate were the same between both types of data, but power decreases for the negatively skewed data and “increases” for the positively skewed data. The increase in power, however, was due to an overestimated mean difference between the focal and reference group, and should not be considered desirable. For the factor mean comparison, the result patterns were consistent across all three types of data. Since the result patterns were highly similar, we report the simulation results for the negatively skewed data in the following and provide the details for positively skewed data and simulated BACS data in the supplemental materials.

**Fig. 3:**
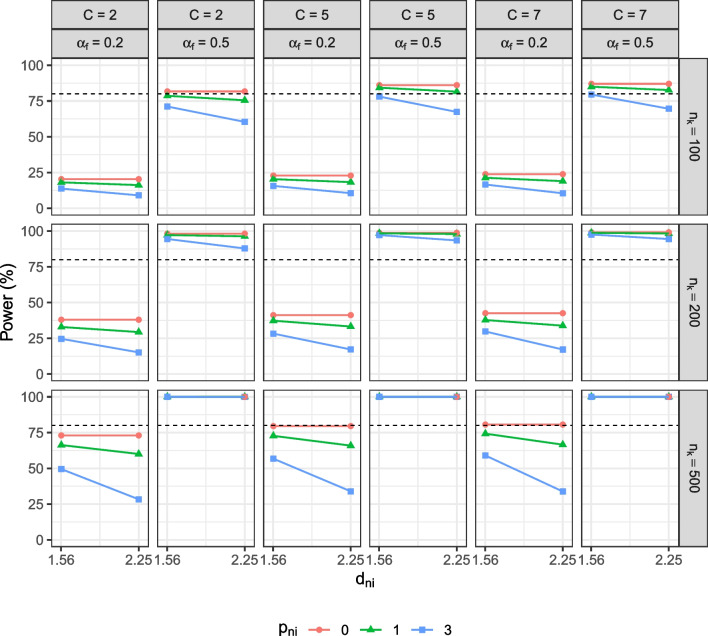
Statistical power of the observed mean comparisons. $n_k$ = group size. *C* = number of response categories. $p_{ni}$ = number of unique factor noninvariant items. $d_{ni}$ = degree of unique factor noninvariance. $\alpha _{f}$ = population factor mean of the focal group. The *dashed line* indicates 80$\%$ power

#### Observed mean comparison

Overall, the effect of unique factor noninvariance on observed mean comparisons was similar for all types of items. As shown in Fig. 2, comparing observed means from data without noninvariant items controlled the type I error rate at the 5% level. However, when unique factor noninvariance was present, observed mean comparisons resulted in an inflated type I error rate. The type I error rate increased with more noninvariant items, a larger degree of unique factor noninvariance, and a larger group size. The type I error rate was similar across item types and was as large as 18.16% for dichotomous items, 18.56% for items with five categories, and 19.52% for items with seven categories when more items ($p_{ni} = 3$) demonstrated a large degree of unique factor noninvariance ($d_{ni} = 1.5^2$).

**Fig. 4:**
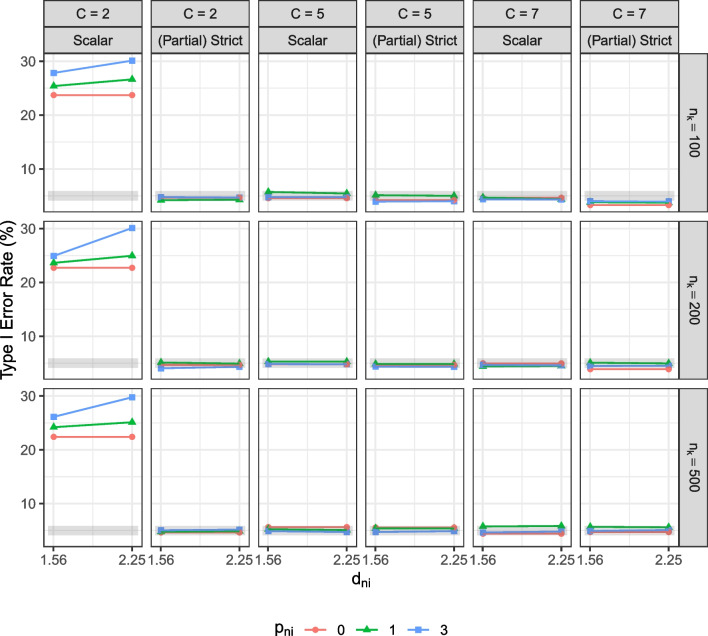
Type I error rate of the factor mean comparisons. $n_k$ = group size. *C* = number of response categories. $p_{ni}$ = number of unique factor noninvariant items. $d_{ni}$ = degree of unique factor noninvariance. $\alpha _{f}$ = population factor mean of the focal group. Scalar = the scalar invariance model. (Partial) Strict = the strict invariance model if all items are invariant or the partial strict invariance model if some items demonstrate unique factor noninvariance. The *shaded area* is the acceptable range of type I error rates, 4.13–5.87%, in this study

**Fig. 5:**
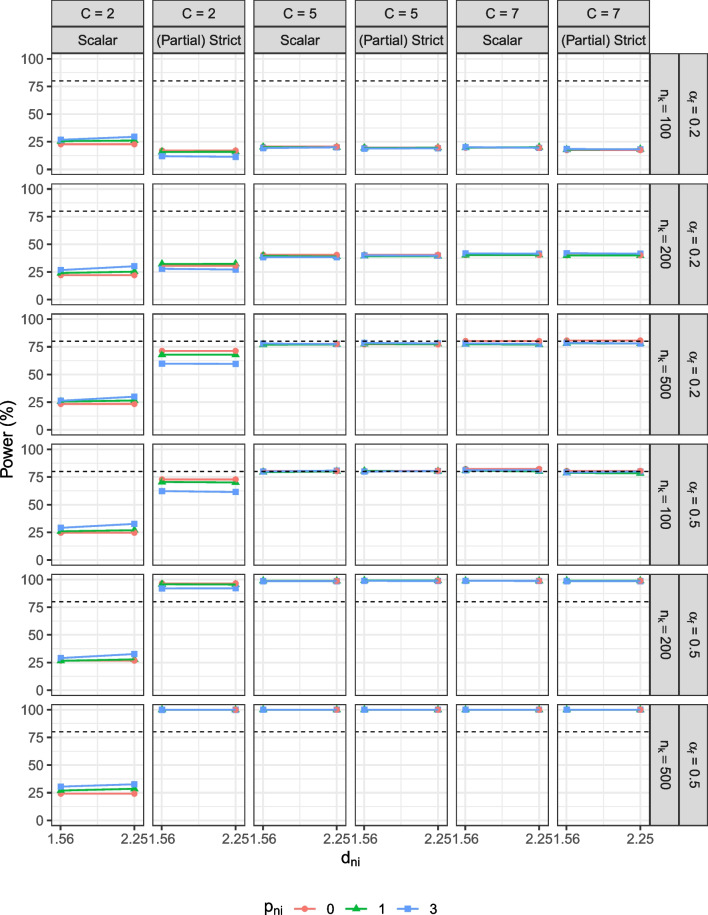
Statistical power of the factor mean comparisons. $n_k$ = group size. *C* = number of response categories. $p_{ni}$ = number of unique factor noninvariant items. $d_{ni}$ = degree of unique factor noninvariance. $\alpha _{f}$ = population factor mean of the focal group. Scalar = the scalar invariance model. (Partial) Strict = the strict invariance model if all items are invariant or the partial strict invariance model if some items demonstrate unique factor noninvariance. The *dashed line* indicates 80$\%$ power

As shown in Fig. 3, compared to the level in the baseline conditions, power dropped as the degree of noninvariance and the number of noninvariant items increased. From the level in the baseline conditions, power decreased from 73% to 28.32%, from 79.52% to 33.92%, and from 80.60% to 33.80% for items with two, five, and seven categories, when the degree of noninvariance and number of noninvariant items were large and the population mean difference was small ($p_{ni} = 3$, $d_{ni} = 1.5^2$, and $\alpha _f = 0.2$).

The raw bias of the observed mean difference is summarized in the supplemental materials. The sample mean difference of observed items underestimated the population mean difference when the data contained noninvariant items. The magnitude of the raw bias increased with more noninvariant items and a larger degree of unique factor noninvariance (e.g., the magnitude of the bias went up to 0.07 when three items had a large degree of noninvariance).

#### Factor mean comparison

Figures 4 and 5 show the results of factor mean comparisons in the scalar and strict/partial strict models. If the simulated data were strict noninvariant, we freely estimated the noninvariant item(s) in the correctly specified partial strict model; otherwise, we evaluated the factor mean difference in the strict model. The model convergence rates were high for items with five response categories (> 99%). For items with seven response categories, the convergence rates were lower (< 80%) when the group size was small $(n_k \!=\! 100)$ but reached high convergence rates (> 99%) when the group size was sufficiently large $(n_k = 200)$. Models failed to converge when there were empty categories in the simulated data, which occurred more often when the group size was small and the number of response categories was large. For dichotomous items, although the convergence rates of the strict/partial strict models were high (> 99%), the convergence rates of the scalar models were low (< 51%) due to model identification issues regardless of sample size conditions.

For dichotomous items, using the strict/partial strict model, factor mean comparisons resulted in type I error rates within the acceptable range. Power was low in conditions with a small group size and a small population factor mean difference ($$n_k = 100$$, $$\alpha _{f} = 0.2$$) but increased as the group size and/or the mean difference were larger. By contrast, using the scalar model, the type I error rate was substantially outside the acceptable range and was highest (30.12%) when the group size was small and more items demonstrated a large degree of unique factor noninvariance $$(n_k = 100$$, $$(p_{ni} = 3$$, $$d_{ni} = 1.5^2)$$. Power in the scalar model was low even when the group size and the population factor mean difference were large $$(n_k = 500$$, $$\alpha _{f} = 0.5)$$. Regardless of whether the simulated data were strict invariant, using the scalar model consistently led to inflated type I error rates and reduced power. On the other hand, for ordered-polytomous items with five or seven categories, using either the scalar or the partial strict invariance model resulted in a type I error rate within the acceptable range and similar power for all conditions.

The supplemental materials include a summary table that shows the raw bias and standard error of the factor mean difference. For dichotomous items, the raw biases and the standard errors were substantially larger in the scalar model than in the strict/partial strict model. Although both the raw biases and the standard errors decreased as the group size increased, they converged to zero more slowly in the scalar model than in the strict/partial strict model. This finding explains the high type I error rate and low power issues in the scalar model for dichotomous items. For ordered-polytomous items, the raw biases were close to zero in both the scalar and strict/partial strict models, and the standard errors were the same between the two models across all conditions.

#### Summary

The results of our simulation study show that different levels of invariance were required for comparing the observed means or factor means with dichotomous or ordered-polytomous items. For all types of ordered-categorical items, valid observed mean comparisons required full strict invariance. Unique factor noninvariance led to biases and erroneous inferences in the observed mean differences between groups for all types of simulated data. For factor mean comparisons, using both scalar and strict/partial strict models yielded similar results for ordered-polytomous items. However, for dichotomous items, comparing factor means in the scalar model consistently resulted in a higher type I error rate, lower power, higher bias, and higher standard error than the strict/partial strict model across conditions.

## Tutorial on measurement invariance testing for ordered-categorical items

In the following tutorial, we aim to demonstrate the MI testing procedure with ordered-categorical items and illustrate mean comparisons when a subset of the items fails the invariance assumptions (i.e., partial invariance). Although the previous literature has discussed the procedure for testing configural, metric, and scalar invariance with ordered-categorical items (e.g., Bowen & Masa, 2015; Svetina et al. , 2019), we extend the demonstration to the test of strict invariance and the search for partial invariance when a few items exhibit threshold or unique factor noninvariance.

The tutorial follows the identification conditions proposed by Millsap and Tein (2004) and Liu et al. (2017). While there are alternative procedures for MI testing with ordered-categorical items, such as Wu and Estabrook (2016) and Svetina et al. (2019), regardless of identification conditions, the central idea remains that researchers should ensure strict invariance before comparing the observed means with ordered-categorical items and adjust for strict noninvariance to make valid factor mean comparisons with dichotomous items. We provide the *lavaan* syntax for MI testing with ordered-polytomous items in the following. The supplemental materials include the complete R script of this tutorial with both ordered-polytomous and dichotomous items.

We used the same example as in previous sections: the seven-item Helpful subscale of BACS developed by Sharman et al. (2019). The data were collected from a sample of 210 college students aged between 17 and 48 ($71.4\%$ female; $M_{age} = 20.18$, $SD = 4.79$). The reliability of the subscale is high with Cronbach’s $\alpha =$ 0.91. Our goal was to examine whether there is a gender difference in the helpful beliefs about crying. For replicability, we use the following syntax to import the data provided by Sharman et al. (2019) and select only relevant variables, including the grouping variable and the seven items in the Helpful subscale:
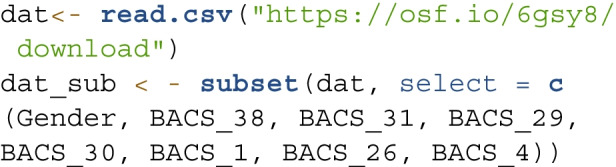


We begin this tutorial by testing the unidimensionality assumption, which is a prerequisite for the use of the observed mean of a psychological scale (McNeish & Wolf, 2020; Widaman & Revelle, 2022) and a one-factor model.^8^ Unidimensionality denotes that a single dimension underlies a set of items and can be evaluated with statistical methods such as the scree plot (Cattell, 1966), parallel analysis (Horn , 1965; Humphreys & Montanelli, 1975; Velicer , 1976), and the Hull method (Lorenzo-Seva et al., 2011). We briefly illustrate the test for the unidimensionality assumption with parallel analysis and refer interested readers to Bandalos (2018) for a comprehensive discussion of other methods. To perform parallel analysis on the Helpful subscale, we utilize the fa.parallel() function in the *psych* package (Revelle, 2022).
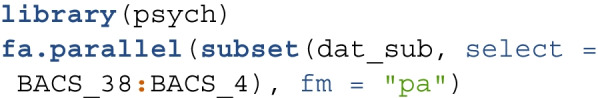


The first three eigenvalues from the parallel analysis are 4.24, 0.16, and 0.09, where the first eigenvalue was substantially larger than the subsequent eigenvalues. The result supports the undimensionality assumption that there is one factor underlying the seven items of the Helpful subscale.

In this tutorial, we follow the MI testing procedure discussed in Liu et al. (2017) and sequentially evaluate configural, metric, scalar, and strict invariance. The configural model is identified by fixing the common factor variance to 1 for the reference group and freely estimate all loadings Wu and Estabrook (2016).^9^ For ordered-polytomous items, the configural model has additional identification constraints as follows (Liu et al. , 2017, p. 494):

1. Fix the latent intercepts $\nu _j$ to 0 across groups.
2. For each of $m$ common factors, select an observed item as the marker variable, and fix the loading of this marker variable to equality across all groups.
3. In one group (i.e., the reference group), fix the common factor mean $\alpha _k$ to 0 and the unique factor variances $\theta _k$ to 1. For the remainder of the groups, freely estimate the unique factor variances.
4. Fix one threshold for each item across groups. For the marker variable, additionally fix a second threshold.

We start with identifying the marker variable, which should have an invariant loading between groups, at least two invariant thresholds, and a meaningful metric or a high factor loading (Liu et al. , 2017). We fit a single-group one-factor model to the data and identify BACS_38 as a candidate item, which has the highest factor loading. With BACS_38 as the marker variable, we continue the MI testing procedure and examine if this item has invariant loadings and/or thresholds. If invariance fails in this item, we return to the beginning and select another candidate item as the marker variable. This process is repeated until a marker variable has been identified.

To identify the set of thresholds to constrain, we initially fix the first threshold of all items to be equal between groups and then examine whether the selected thresholds are invariant in the metric model. If invariance holds for these thresholds, we will proceed to the next stage of invariance testing; otherwise, we return to the beginning and repeat the process with another set of thresholds (Liu et al., 2017).

In all invariance models, we use the cfa() function to perform MG-CFA along with specifying the grouping variable in group = "Gender". To account for the ordered-categorical nature of the data, we specify the items as ordered and the estimation method as "WLSMV" with "theta" parameterization. Depending on the specific model, mod refers to the corresponding *lavaan* model syntax, which is available in the supplemental materials. The syntax of the configural model is as follows:
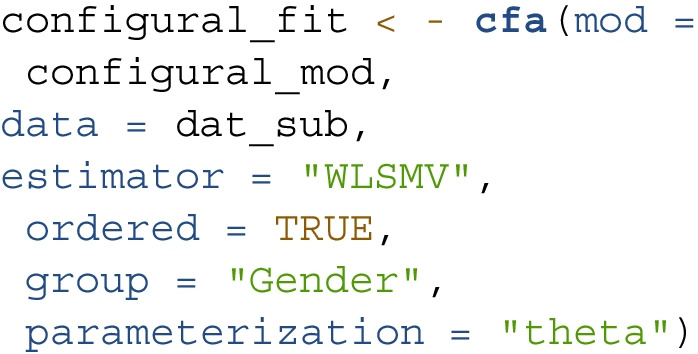


This configural model has an acceptable fit, $\chi ^2(28) = 56.56$, $p = .001$, RMSEA = 0.10, 95% CI [0.06, 0.14], CFI = 0.99, SRMR = 0.04.

We then move on to assess metric invariance, which has the same identification constraints as the configural model, except that it includes additional equality constraints on the loadings across groups. This metric model has an acceptable fit, $\chi ^2(34) = 61.25$, $p = .003$, RMSEA = 0.09, 95% CI [0.05, 0.12], CFI = 0.99, SRMR = 0.04. The modification indices (see syntax below) suggest the loadings and thresholds of item BACS_38 are invariant, as well as the first threshold of all items.
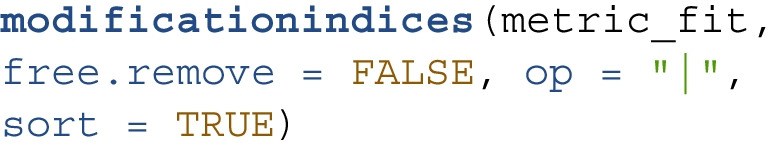


Thus, we confirm the initial identification constraints for the configural model, i.e., fixing the first threshold of all items equal between groups and using BACS_38 as the marker variable. The chi-square difference test is statistically nonsignificant (syntax provided below), scaled $\Delta \chi ^2(6) = 9.13$, $p = .166$, suggesting insufficient evidence that the loadings are noninvariant.^10^
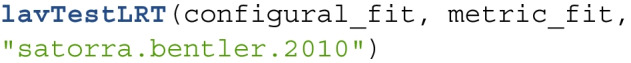


Next, we move on to the scalar model which further constrains all thresholds to be equal between groups in addition to the constraints in the metric model. The scalar model has an acceptable fit, $\chi ^2(54) = 101.91$, *p* < .001, RMSEA = 0.09, 95%CI [0.06, 0.12], CFI = 0.99, SRMR = 0.04, but is significantly different from the metric model, scaled $\Delta \chi ^2(20) = 43.41, p = .002$, indicating that some thresholds are noninvariant.

Since full threshold invariance failed, the unconstrained thresholds for all items must be tested sequentially to identify the noninvariant threshold(s). This sequential specification search has been found to perform well in controlling false positive rates (Yoon & Kim, 2014). As the modification index suggests that the first threshold of BACS_30 is noninvariant, we free this threshold and use the resulting model as the partial scalar model. The partial scalar model has an acceptable fit $(\chi ^2(53) = 91.11)$, $(p = .001)$, $\text {RMSEA} = 0.08, 95\%~\text {CI} {[}0.05, 0.11{]}, \text {CFI} = 0.99, \text {SRMR} = 0.04$, and does not fit worse than the metric model, scaled $\Delta \chi ^2(19) = 32.15$, $p = .030$. Thus, we proceed to the partial strict invariance model, which constrains the unique factor variances to be equal in the items that have invariant thresholds in the partial scalar model.

The fit of the partial strict model is similar to that of the partial scalar model, scaled $\Delta \chi ^2(6) = 15.76$, $p = .015$. Therefore, the final model is a partial strict invariant model with the first threshold and unique factor variance of the item BACS_30 freed. In other words, items BACS_38, BACS_31, BACS_29, BACS_1, BACS_26, BACS_4 are strict invariant, whereas item BACS_30 is metric invariant. This final model has acceptable fit, $\chi ^2(59) = 106.03$, $p <.001$, $\text {RMSEA} = 0.09, 95\%~\text {CI} {[}0.06, 0.11{]}, \text {CFI} = 0.99, \text {SRMR} = 0.05$.

As only partial strict invariance holds, we recommend not comparing observed means of this subscale between groups. Instead, we can compare the factor means in the partial scalar or partial strict model and use the following command, for example, to examine the factor mean difference in the partial strict model:
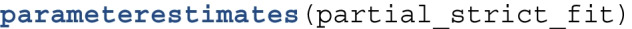


The factor mean difference is statistically significant in both the partial scalar model, $-.63, 95\% \text { CI }[-0.99, -0.29]$, and the partial strict model, $-.64, 95\% \text { CI }[-0.99, -0.28]$. For ordered-polytomous items, factor mean comparisons are valid in both the scalar/partial scalar and strict/partial strict models. For dichotomous items, however, we recommend researchers compare factor means in only the strict/partial strict model, as suggested in the simulation results.

One thing to note is we used the sequential approach of testing proposed by Yoon and Millsap (2007), which does not guarantee to yield the true model when a large number of items violate the MI assumption (Yoon & Kim, 2014). Since we did not find evidence of noninvariance for all items except item BACS_30, we believe the results given by this sequential approach are valid. Further details on the comparisons of sequential approach versus nonsequential approach can be found in Yoon and Kim (2014) and Pohl et al. (2021).

## Discussion

The literature lacks consensus about the necessary condition for valid mean comparisons with ordered-categorical items (Pendergast et al., 2017). On one hand, generalized from the literature for continuous items, some authors assumed that strict invariance is optional for ordered-categorical items when comparing factor means (e.g., Bauer , 2017; Bovaird & Koziol, 2012; Putnick & Bornstein, 2016), as well as when comparing observed means across groups (e.g., Svetina et al. , 2019). Therefore, strict invariance has rarely been tested in published research (Svetina et al., 2019), as observed in the brief review of the present paper on MI testing with ordered-categorical items. On the other hand, Liu et al. (2017) argued that strict invariance is needed for valid observed mean comparisons with ordered-categorical items. Given the inconsistent recommendations in the literature, the aim of the present paper was to revisit the question: Is strict invariance a prerequisite for valid group comparisons of observed means and factor means with dichotomous and ordered-polytomous items?

For observed mean comparisons, the present study echoes the argument of Liu et al. (2017) that valid group comparisons require ordered-categorical items to achieve full strict invariance: invariance of loadings, thresholds, intercepts, and unique factor variances. In the simulation study, we found that the observed mean difference had increased bias and inflated type I error rate as the number of unique factor noninvariant items and the degree of noninvariance increased. We note that the impact of unique factor noninvariance could be worse than what is shown in the simulation study. For example, if the items were simulated with a stronger skewness of -2, the type I error rate would increase to more than 40% and power would decrease by more than 60 percentage points for all item types. Furthermore, as a function of group size, the type I error rate could also reach more than 50% for all item types when we increased the group size to 2000. We report details of additional analyses in the supplemental materials.

Relatedly, the distributions of the observed responses also impact the magnitude of the bias. As shown in Appendix A, the impact of unique factor noninvariance reduces with a less skewed (i.e., more symmetric) distribution. Stated differently, ordered-categorical items with a more symmetric distribution are less influenced by unique factor noninvariance in observed mean difference and behave more similarly as continuous items. This is in line with previous studies, which showed that continuous methodology can outperform categorical methodology for ordered-categorical items with a symmetric distribution (e.g., Rhemtulla et al. , 2012; Sass et al. , 2014).

The simulation study showed that for dichotomous items, factor mean comparisons are valid only in the correctly specified strict/partial strict invariance model; for ordered-polytomous items, such comparisons are valid in both the correctly specified scalar/partial scalar and strict/partial strict invariance models. Consistent with the past literature (e.g., Wu & Estabrook, 2016), scalar invariance (i.e., invariance of loadings and thresholds) effectively equates the scale of the latent responses with the latent common factor for ordered-polytomous items. As such, the factor mean difference in the scalar model accurately reflects the group difference in the latent common factor. By contrast, dichotomous items contain fewer response categories and reduced information than ordered-polytomous items, resulting in unidentified parameters when the unique variances freely vary between groups. As confirmed in the simulation study, the factor mean difference in the scalar model consistently had inflated type I error rates, lower power, higher biases, and higher standard errors than in the strict/partial strict model. Such biases are present in the scalar model even when the data are strict invariant.

In summary, for dichotomous items, we strongly advise testing strict invariance prior to any form of mean comparisons. If full strict invariance holds, one can compare the observed means or compare the factor means in the strict model, but not in the scalar model. If strict invariance fails, one should establish a partial strict invariance model to compare the factor means with dichotomous items. For ordered-polytomous items, if the goal is to compare observed means, we suggest the support of full strict invariance. Otherwise, factor mean comparison is valid in either the correctly specified scalar or strict model.

### Limitations and future research directions

The current paper fills the gap in the literature about the necessary invariance conditions for factor and observed mean comparisons with ordered-categorical items. However, we have not examined the impact of unique factor noninvariance on more complex analyses, such as regression and longitudinal analysis, with ordered-categorical items. It is relatively straightforward to generalize mean comparison to a regression model with the group membership as the only predictor and the latent common factor of interest as the outcome variable measured by a set of ordered-categorical items, as these are equivalent analyses. However, when the regression model includes an additional latent predictor that is measured by another set of ordered-categorical items, it is unclear whether unique factor noninvariance would bias the regression slopes. While past research has investigated the impact of loading and intercept noninvariance on regression slopes (Chen , 2008; Hsiao & Lai, 2018), similar future research should similarly examine the effect of unique factor noninvariance on regression slopes.

Furthermore, the current paper focused on the invariance of unique factor variances; however, whether the results generalize to the invariance of unique factor covariances requires further investigation. Liu et al. (2017) mathematically showed that observed mean comparisons require invariance of unique factor variances as well as covariances. Future research could evaluate the impact of which noninvariance in unique covariances on statistical inferences and parameter estimations of observed and factor mean comparisons.

## Open Practices Statement

Simulation codes and data are openly available on the project’s GitHub page (https://github.com/winniewytse/micat-supp).

## Figures and Tables

**Fig. 6:**
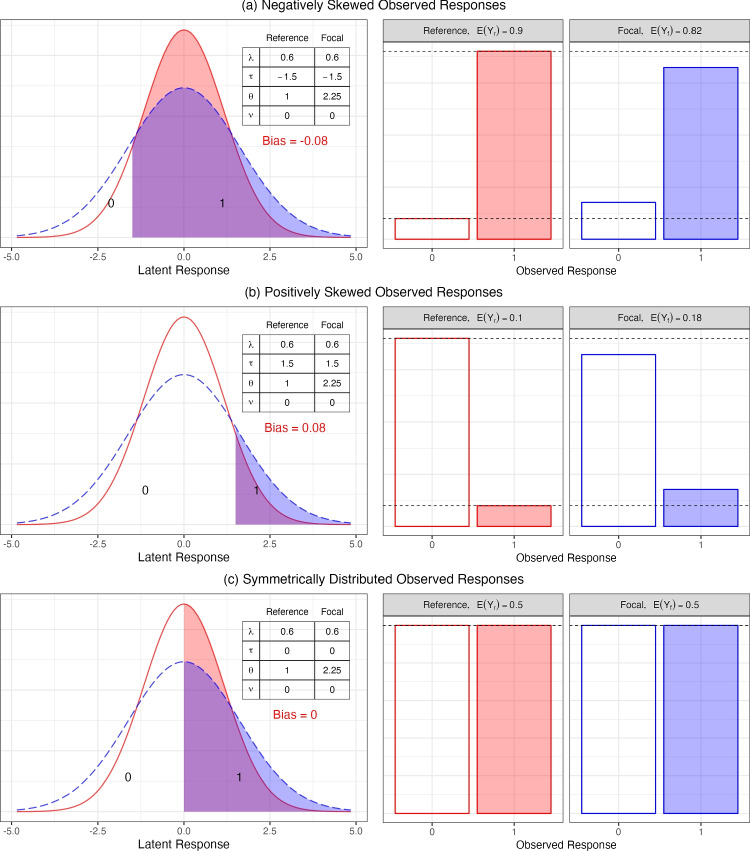
Illustration of biases due to unique factor noninvariance. Both groups share the same loading ($\lambda$), threshold ($\tau$), intercept ($\nu$), factor mean ($\alpha = 0$), and factor variance ($\psi = 1$), but differ in unique variances ($\theta _r \ne \theta _f$). E(Y) denotes the observed group mean. Bias indicates the observed mean difference due to unique factor noninvariance as the population mean difference is assumed 0. The observed response distribution is negatively skewed when $\tau = -1.5$ (**a**), positively skewed when $\tau = 1.5$ (**b**), symmetric when $\tau = 0$ (**c**)

**Fig. 7:**
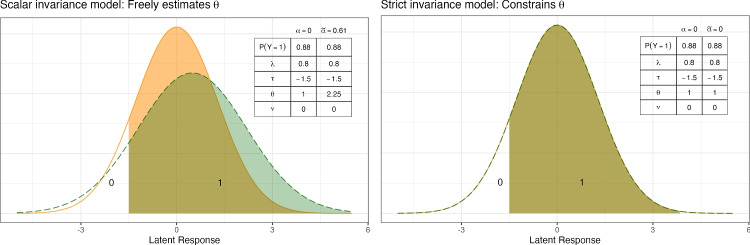
Indeterminacy in the factor mean of a dichotomous item. All *curves* show the latent response distributions of a dichotomous item, with categories of 0 (below $\tau$) and 1 (above $\tau$). Assume that the population factor mean is $\alpha = 0$ (*orange*, *solid curve*), and the probability of endorsing 1 is $P(Y = 1) = 88\%$. A scalar invariance model (*left panel*), which freely estimates the unique variance to be $\tilde{\theta } = 2.25$, yields a factor mean estimate of $\tilde{\alpha } = 0.61$ (*purple*, *dashed curve*). A strict invariance model (*right panel*), which constrains the unique variance to be $\tilde{\theta } = 1$, yields a factor mean estimate of $\tilde{\alpha } = 0$ (*purple*, *dashed curve*). $\lambda$ = factor loading. $\tau$ = threshold. $\theta$ = unique factor variance. $\nu$ = intercept

## Observed mean comparison

When items are continuous and normally distributed, establishing scalar invariance allows group comparisons with means of both the factor means and observed means (Millsap, 2011). Specifically, under the assumption that $E(\varepsilon _j) = 0$ for all (j)s, from (2), the expected value of the observed items (i.e., observed mean), is

A.1 $$\begin{aligned} {{\,\textrm{E}\,}}(Y_{ij} | \eta _i) = \nu _j + \lambda _j \eta _i, \end{aligned}$$

which is not a function of the unique factor $\varepsilon$. This shows that having invariant intercepts and factor loadings secures equality of the scales of observed continuous items across groups. In other words, when scalar invariance is achieved, if any individuals from the two groups have the same factor means, they should also have the same observed means at the population level. In this case, differences in the observed means accurately reflect differences in the latent construct.

However, with regard to the expected value of observed ordered-categorical items, only when strict invariance holds (i.e., $\nu _j$, $\tau _j$, $\lambda _j$, and $\theta _j$ are invariant across groups) are changes in the expected values of the observed ordered-categorical items solely due to changes in the latent construct between groups (Liu et al., 2017). That is, differences in the expected values of the observed ordered-categorical items are unbiased estimates of differences in the latent construct only if strict invariance holds. Liu et al. (2017) detailed the mathematical support for the necessity of strict invariance for valid observed score comparisons of ordered-categorical items. In the following we illustrate the cases of dichotomous and ordered-polytomous variables separately.

### Observed means of dichotomous items *(C = 2)*

First let $Y$ be a dichotomous item, which follows a Bernoulli distribution, conditioned on $\eta _i$. The expected value of $Y$, given $\eta = \eta _0$ for an arbitrary $\eta _0$ value, is:

A.2 $$\begin{aligned} {{\,\textrm{E}\,}}(Y_{ij}|\eta _i&= \eta _0)= P(Y_{ij} = 1 | \eta _0) = P(Y^*_{ij}>\tau _{j} | \eta _0) \\ \nonumber&= P\left( \frac{Y^*_{ij} - \nu _j - \lambda _j \eta _0}{\sqrt{\theta _j}}>\frac{\tau _{j} - \nu _j - \lambda _j \eta _0}{\sqrt{\theta _j}}\right) \\ \nonumber&= P\left( Z>\frac{\tau _{j} - \nu _j - \lambda _j \eta _0}{\sqrt{\theta _j}}\right) \\ \nonumber&= 1 - \Phi \left( \frac{\tau _{j} - \nu _j - \lambda _j \eta _0}{\sqrt{\theta _j}}\right) , \end{aligned}$$

where $Z$ is a standard normal variable and $\Phi (\dot{)}$ is the standard normal cumulative distribution function (cdf). The marginal distribution of the latent responses, $Y^*$, has an expected value of $\text {E}(Y^*_{ij}) = \nu _i + \lambda _j \alpha$ and a variance of $\text {Var}(Y^*_{ij}) = \lambda _j^2 \psi + \theta _j$. Thus, the marginal distribution of $Y$ has an expected value of

A.3 $$\begin{aligned} {{\,\textrm{E}\,}}(Y_{ij}) = 1 - \Phi \left( \frac{\tau _{j} - \nu _j - \lambda _j \alpha }{\sqrt{\lambda _j^2 \psi + \theta _j}}\right) . \end{aligned}$$

As illustrated, the expected value of $y$ is a function of not only $\tau _j$, $\nu _j$, and $\lambda _j$, which are part of the assessment of scalar invariance, but also of $\theta _j$, which is only examined in the strict invariance model.

### Observed means of ordered-polytomous items ($C > 2$)

Similarly, when $Y$ is an ordered-polytomous variable with $C$ categories for $C > 2$, it follows a categorical distribution, conditioned on $\eta _i$. Let $P^{(c)}_{ij} = P(Y^*_{ij} \le \tau ^{(c)} | \eta _i = \eta _0)$ be the probability that $Y_{ij} < c$. The expected value of $Y$ is (Liu et al., 2017):

A.4 $$\begin{aligned} \textrm{E}(Y_{ij} | \eta _i = \eta _0)&= \sum _{c = 0}^{C-1} c P(Y_{ij} = c | \eta _i = \eta _0) \nonumber \\&= \sum _{c = 1}^{C - 2} c P(\tau _{j}^{(c)}< Y^*_{ij} \le \tau _{j}^{(c + 1)} | \eta _i = \eta _0) \nonumber \\ {}&\quad + (C - 1) P(Y^*_{ij}> \tau _{j}^{(C-1)} | \eta _i = \eta _0) \\ \nonumber&= [P^{(2)}_{ij} - P^{(1)}_{ij}] + 2 [P^{(3)}_{ij} - P^{(2)}_{ij}] +\ldots \\ \nonumber&\quad + (C - 2) [P^{(C - 1)}_{ij} - P^{(C - 2)}_{ij}] \\ \nonumber&\quad + (C - 1) [1 - P^{(C - 1)}_{ij}] \\ \nonumber&\quad = (C - 1) - \sum _{c = 1}^{C - 1} P^{(c)}_{ij} \\ \nonumber&\quad = (C - 1) - \sum _{c = 1}^{C - 1} \Phi \left( \frac{\tau _{j}^{(c)} - \nu _j - \lambda _j \eta _0}{\sqrt{\theta _j}}\right) , \end{aligned}$$

where the last step follows from the derivation in the dichotomous case. In a similar fashion, the marginal distribution of $Y$ has an expected value of

A.5 $$\begin{aligned} \textrm{E}(Y_{ij})&= (C - 1) - \sum _{c = 1}^{C - 1} \Phi \left( \frac{\tau _{j}^{(c)} - \nu _j - \lambda _j \alpha }{\sqrt{\lambda _j^2 \psi + \theta _j}}\right) . \end{aligned}$$

Once again, the expected value of the observed item score is a function of $\theta _j$. This shows that group comparisons with the observed ordered-categorical items are only valid when all $\nu _j$, $\tau _j$, $\lambda _j$, and $\theta _j$ are invariant.

### Bias in observed mean difference due to unique factor noninvariance

Consider that $Y$ is an ordered-categorical item with invariant loadings ($\lambda _r = \lambda _f$), thresholds ($\tau _r^{(c)} = \tau _f^{(c)}$), and intercepts ($\nu _r = \nu _f$), but noninvariant unique factor variances ($\theta _r \ne \theta _f$) between two groups, reference and focal. Suppose that the two groups share the same mean ($\alpha _{r} = \alpha _{f} = \alpha$) and variance ($\psi _r = \psi _f = \psi$) in a latent construct. If the observed scores accurately reflect their mean standings in the latent construct, the observed mean difference between the two groups should be equal to zero. However, the unique factor noninvariance induces an observed mean difference of

A.6 $$\begin{aligned} {{\,\textrm{E}\,}}(Y_{ijr}) - {{\,\textrm{E}\,}}(Y_{ijf})&=\left[ (C-1) - \sum _{c = 1}^{C - 1} \Phi \left( \frac{\tau _{j}^{(c)} - \nu _j - \lambda _j \alpha }{\sqrt{\lambda _j^2\psi + \theta _{jr}}}\right) \right] \\ \nonumber&\quad - \left[ (C - 1) - \sum _{c = 1}^{C - 1} \Phi \left( \frac{\tau _{j}^{(c)} - \nu _j - \lambda _j \alpha }{\sqrt{\lambda _j^2\psi + \theta _{jf}}}\right) \right] \\ \nonumber&=\sum _{c = 1}^{C - 1} \Phi \left( \frac{\tau _{j}^{(c)} - \nu _j - \lambda _j \alpha }{\sqrt{\lambda _j^2\psi + \theta _{jf}}}\right) \\ \nonumber&\quad - \sum _{c = 1}^{C - 1} \Phi \left( \frac{\tau _{j}^{(c)} - \nu _j - \lambda _j \alpha }{\sqrt{\lambda _j^2\psi + \theta _{jr}}}\right) , \\ \nonumber \end{aligned}$$

which is non-zero unless $\theta _{jf} = \theta _{jr}$ or $\tau ^{(c)}_j - \nu _j - \lambda _j \alpha = 0$.

The distribution shape of observed responses generally affects the direction and magnitude of bias due to unique factor noninvariance, which can be derived using Eq. A.6. As noted above, the bias will be zero when $\tau _j - \nu _j - \lambda _j \alpha = 0$. The bias will also be zero if the observed distribution is symmetric (e.g., with an equal probability of endorsing Category 0 or 1 for a dichotomous item). Figure 6 illustrates an example of biases in observed mean difference for a dichotomous item with different shapes of observed distributions.

## Factor mean comparison

Here we provide the mathematical support for that factor mean comparisons with dichotomous items require the use of strict or partial strict invariance model, whereas such comparisons with ordered-polytomous items are permissible in the scalar invariance model.

### Factor means of dichotomous items ($C = 2$)

Consider a dichotomous item with invariant loadings ($\lambda _r = \lambda _f$), thresholds ($\tau _r = \tau _f$), intercepts ($\nu _r = \nu _f$), and unique factor variances ($\theta _r = \theta _f$), for two groups, reference and focal. That is, the item achieves strict invariance. Also, assume that both groups have the same population factor mean and variance, i.e., $\alpha _r = \alpha _f$ and $\psi _r = \psi _f$. The population mean difference is hence zero. With equality constraints on loadings, intercepts, and thresholds, under typical identifications (i.e., $\nu _r = \nu _f = 0$) and theta parameterization, the scalar model

1. estimates $\lambda _r$ and fixes $\lambda _f$ to be the same as $\lambda _r$;
2. estimates $\tau _r$ and fixes $\tau _f$ to be the same as $\tau _r$;
3. fixes $\theta _r$ to 1 and estimates $\theta _f$; and
4. fixes $\alpha _r = 0$ and estimates $\alpha _f$.

Suppose that the parameter estimates of the reference group recovers the population parameters. Without loss of generality, we additionally constrain $\psi _r = \psi _f$ in the model to examine the relationship between $\alpha$ and $\theta$. For the focal group, the scalar model constrains $\lambda _r = \lambda _f$ and $\tau _r = \tau _f$, leaving $\theta _f$ and $\alpha _f$ to be freely estimated. In the following, we focus on the focal group and drop the subscripts of the parameters. For dichotomous items in the item-factor model, parameters are estimated by equating the univariate proportion of participants endorsing Category 1, $P(Y=1)$, to the model implied proportion. That is

B.1 $$\begin{aligned} P(Y=1) = 1 - \Phi \left( \frac{\tau - \lambda \alpha }{\sqrt{\lambda ^2 \psi +\theta }} \right) . \end{aligned}$$

For the focal group in the scalar model, while $\tau , \lambda ,$ and $\psi$ are known by the model constraints, $\alpha$ and $\theta$ remain undetermined. In other words, there exists at least one other set of estimated factor mean and unique variance, $\tilde{\alpha }$ and $\tilde{\theta }$, which yields the same model implied statistic, $P(Y=1)$, for this item. The indeterminacy of the factor mean in the scalar model can be expressed as follows,

B.2 $$\begin{aligned} P(Y=1) = 1 - \Phi \left( \frac{\tau - \lambda \alpha }{\sqrt{\lambda ^2 \psi +\theta }} \right)&= 1 - \Phi \left( \frac{\tau - \lambda \tilde{\alpha }}{\sqrt{\lambda ^2 \psi +\tilde{\theta }}} \right) \\ \nonumber \frac{\tau - \lambda \alpha }{\sqrt{\lambda ^2 \psi +\theta }}&= \frac{\tau - \lambda \tilde{\alpha }}{\sqrt{\lambda ^2 \psi +\tilde{\theta }}}\\\nonumber \tilde{\alpha }&=\alpha \sqrt{\frac{\lambda ^2 \psi + \tilde{\theta }}{\lambda ^2 \psi + \theta }} \\\nonumber&\quad + \frac{\tau }{\lambda }\left( 1 - \sqrt{\frac{\lambda ^2 \psi + \tilde{\theta }}{\lambda ^2 \psi + \theta }}\right) . \end{aligned}$$

Notice that the factor mean is uniquely identified, $\tilde{\alpha }= \alpha$, only when the unique variance is fixed at some value such that $\tilde{\theta }= \theta$. As shown in the derivation, more than one sets of the factor mean and unique factor variance estimates correspond to the same model-implied statistic, indicating the same the latent response distribution ($Y^*$). As such, the factor mean is not uniquely identified when unique factor variances are freely estimated in a dichotomous item.

To illustrate, suppose that the population parameters of the dichotomous item are $\lambda _r = \lambda _f = .8$, $\tau _r = \tau _f = -1.5$, $\nu _r = \nu _f = 0$, $\theta _r = \theta _f = 1$, $\alpha _r = \alpha _f = 0$, and $\psi _r = \psi _f = 1$. For example, 88% participants in the focal group endorsed Category 1. As shown in Fig. 7, in the scalar model, a set of solution $\tilde{\alpha }_f = .61$ and $\tilde{\theta }_f = 2.25$ yields the same univariate proportion, $P(\tilde{Y} = 1) = .88$, as does another set of solution that aligns with the population parameters $\alpha _f = 0$ and $\theta _f = 1$, $P(Y = 1) = .88$. Whereas the population factor mean is 0, the scalar model gives an estimated factor mean of $.61$ for the focal group by freeing its unique variance, artificially inflating the factor mean difference in this example. Contrarily, the strict model constrains the unique variance of the focal group to be the same as the unique variance of the reference group. With the equality constraint of the unique variances between groups, the factor mean is uniquely identified and accurately recovers the population factor mean and hence the population factor mean difference.

### Factor means of ordered-polytomous items ($C > 2$)

The indeterminacy issue of the factor mean in dichotomous items does not generalize to ordered-polytomous items, which have more response categories and provide more information than dichotomous items. Consider an ordered-polytomous item with three response categories: $\{0, 1, 2\}$. Similarly, in the item-factor model, the parameters are estimated by equating the univariate proportions to the model implied proportions of each response category, given by the following set of equations

B.3 $$\begin{aligned} {\left\{ \begin{array}{ll} P(Y=1) = \Phi \left( \frac{\tau ^{(2)} - \lambda \alpha }{\sqrt{\lambda ^2 \psi +\theta }} \right) - \Phi \left( \frac{\tau ^{(1)} - \lambda \alpha }{\sqrt{\lambda ^2 \psi +\theta }} \right) \\ P(Y=2) = 1 - \Phi \left( \frac{\tau ^{(2)} - \lambda \alpha }{\sqrt{\lambda ^2 \psi +\theta }} \right) . \\ \end{array}\right. } \end{aligned}$$

For the focal group in the scalar model, $\tau ^{(1)}, \tau ^{(2)}$, and $\lambda$ are known as they are set to be equal to the parameter estimates of the reference group. Suppose that we constrain $\psi$ to be equal between groups such that it is also known for the focal group. This leaves two unknowns, $\alpha$ and $\theta$, to be solved by two equations. Therefore, the scalar model can uniquely identify the factor mean without having to constrain the unique variance. Specifically, the scalar model sufficiently equates the location and scale of the two groups with the equality constraints on loadings and thresholds. With additional equality constraints on the unique variances, the strict model yields comparable results to those of the scalar model, as confirmed in the simulation study. Note that an item with more than three response categories has more than two equations to solve the two unknowns, $\alpha$ and $\theta$. As such, the factor mean is still uniquely identified.

## Footnotes

1 We refer to the unique factor covariance matrix as “unique factor variance” or “unique variance” for short. We indicate the invariance of unique factor variances and covariances through the “unique factor invariance”.

2 We refer to the observed means as the unweighted sum or mean scores of the observed items and the factor means as the means of the latent common factors in a multigroup confirmatory factor analysis.

3 An alternative approach is developed within the item response theory (IRT) framework (Penfield & Lam, 2005; Teresi , 2006) to test MI, also known as differential item functioning, for ordered-categorical items. While IRT is beyond the scope of this paper, we refer interested readers to other excellent sources on this approach (Meade and Lautenschlager , 2004; Tay et al. , 2015).

4 DWLS and its variants (e.g., unweighted least squares [ULS], weighted least square mean and variance adjusted [WLSMV]) are estimators that allow unique variances to freely vary. Other estimation methods, such as maximum likelihood (ML) and ML estimation with robust standard errors (MLR), typically fix the unique variances to 1 (Asparouhov & Muthén, 2020). As such, the equality of unique variances is already assessed in earlier stages of invariance models, and achieving scalar invariance with these estimators implies equality of loadings, thresholds, and unique variances.

5 An alternative is to apply a logit link by assuming that $\varepsilon$ follows a standard logistic distribution (see Wirth & Edwards, 2007). The analytic results in the current study also apply to the logistic parameterization.

6 McNeish and Wolf (2020) and McNeish (2022) discussed that using sum (or mean) scores require strict assumptions such as equal factor loadings across items, whereas Widaman and Revelle (2022) argued that using sum scores requires only unidimensionality and avoids indeterminacy issues in factor scores. We recommend consulting these papers for a more in-depth discussion and advise researchers to carefully examine their data to make educated decisions in comparing observed scores or factor scores. We note that whether or not the equal factor loading assumption holds, valid observed mean comparisons require unique factor invariance, as shown in the current simulation study using parameter values that have equal loadings (adopted from Sass et al. , 2014) and unequal loadings (adopted from the BACS empirical data).

7 We have also fit the scalar models using the delta parameterization (provided in supplemental materials), of which the results are consistent with the scalar models using theta parameterization.

8 We thank an anonymous reviewer for suggesting the inclusion of the test for unidimensionality in the tutorial.

9 An alternative and equivalent way of model identification is to fix the loading of an item to 1 and free the common factor variance for the reference group (Liu et al. , 2017; Millsap & Tein, 2004).

10 Because the usual chi-square different test statistics may not follow a $\chi ^2$ distribution, given the distribution of the data, Satorra and Bentler (2001) proposed a scaled chi-square difference test to improve the chi-square approximation. The current study used this scaled chi-square difference test to compare model fit.

## References

[CR1] Asparouhov, T., & Muthén, B.O. (2020). IRT in Mplus (Version 4). http://www.statmodel.com/download/MplusIRT.pdf

[CR2] Avison WR, McAlpine DD (1992). Gender differences in symptoms of depression among adolescents. Journal of Health and Social Behavior.

[CR3] Bandalos DL (2018). Measurement theory and applications for the social sciences.

[CR4] Bauer DJ (2017). A more general model for testing measurement invariance and differential item functioning. Psychological Methods.

[CR5] Birnbaum, A. (1968). Some latent trait models and their use in inferring an examinee’s ability. In *Statistical theories of mental test scores* (pp. 395–479). Addison-Wesley.

[CR6] Bovaird, J. A., & Koziol, N. A. (2012). Measurement models for ordered-categorical indicators. In *Handbook of structural equation modeling* (pp. 495–511). The Guilford Press.

[CR7] Bowen NK, Masa RD (2015). Conducting measurement invariance tests with ordinal data: A guide for social work researchers. Journal of the Society for Social Work and Research.

[CR8] Byrne BM, Shavelson RJ, Muthén BO (1989). Testing for the equivalence of factor covariance and mean structures: The issue of partial measurement invariance. Psychological Bulletin.

[CR9] Cattell RB (1966). The scree test for the number of factors. Multivariate Behavioral Research.

[CR10] Chalmers, R. P., & Adkins, M. C. (2020). Writing effective and reliable Monte Carlo simulations with the SimDesign package. *The Quantitative Methods for Psychology*, *16*(4), 248–280. 10.20982/tqmp.16.4.p248

[CR11] Chen FF (2008). What happens if we compare chopsticks with forks? The impact of making inappropriate comparisons in cross-cultural research. Journal of Personality and Social Psychology.

[CR12] Fitzpatrick KM, Harris C, Drawve G (2020). Living in the midst of fear: Depressive symptomatology among US adults during the COVID-19 pandemic. Depression and Anxiety.

[CR13] Horn JL (1965). A rationale and test for the number of factors in factor analysis. Psychometrika.

[CR14] Horn JL, McArdle JJ (1992). A practical and theoretical guide to measurement invariance in aging research. Experimental Aging Research.

[CR15] Hsiao Y-Y, Kwok O-M, Lai MHC (2018). Evaluation of two methods for modeling measurement errors when testing interaction effects with observed composite scores. Educational and Psychological Measurement.

[CR16] Hsiao Y-Y, Lai MHC (2018). The impact of partial measurement invariance on testing moderation for single and multi-level data. Frontiers in Psychology.

[CR17] Humphreys LG, Montanelli RG (1975). An investigation of the parallel analysis criterion for determining the number of common factors. Multivariate Behavioral Research.

[CR18] Kite BA, Jorgensen TD, Chen P-Y (2018). Random permutation testing applied to measurement invariance testing with ordered-categorical indicators. Structural Equation Modeling: A Multidisciplinary Journal.

[CR19] Lai, M. H. C., Liu, Y., & Tse, W. W.-Y. (2021). Adjusting for partial invariance in latent parameter estimation: Comparing forward specification search and approximate invariance methods. *Behavior Research Methods*, 1–21. 10.3758/s13428-021-01560-2 34236670

[CR20] Liu Y, Millsap RE, West SG, Tein J-Y, Tanaka R, Grimm KJ (2017). Testing measurement invariance in longitudinal data with ordered-categorical measures. Psychological Methods.

[CR21] Liu Y, West SG (2018). Longitudinal measurement non-invariance with ordered-categorical indicators: How are the parameters in second-order latent linear growth models affected?. Structural Equation Modeling: A Multidisciplinary Journal.

[CR22] Lorenzo-Seva U, Timmerman ME, Kiers HAL (2011). The hull method for selecting the number of common factors. Multivariate Behavioral Research.

[CR23] McNeish, D. (2022). Psychometric properties of sum scores and factor scores differ even when their correlation is 0.98: A response to Widaman and Revelle. *Behavior Research Methods*. 10.3758/s13428-022-02016-x 10.3758/s13428-022-02016-x 36394821

[CR24] McNeish D, Wolf MG (2020). Thinking twice about sum scores. Behavior Research Methods.

[CR25] Meade AW, Lautenschlager GJ (2004). A comparison of item response theory and confirmatory factor analytic methodologies for establishing measurement equivalence/invariance. Organizational Research Methods.

[CR26] Mellenbergh GJ (1989). Item bias and item response theory. International Journal of Educational Research.

[CR27] Meredith W (1993). Measurement invariance, factor analysis and factorial invariance. Psychometrika.

[CR28] Meredith W, Teresi JA (2006). An essay on measurement and factorial invariance. Medical Care.

[CR29] Millsap RE (2011). Statistical approaches to measurement invariance.

[CR30] Millsap RE, Tein J-Y (2004). Assessing factorial invariance in ordered-categorical measures. Multivariate Behavioral Research.

[CR31] Muthén, B.O. (2002). Latent variable analysis with categorical outcomes: Multiple-group and growth modeling in Mplus. *Version 5. Technical Report*, 23.

[CR32] Muthén, L.K., & Muthén, B.O. (1998–2017). Mplus user’s guide (8th ed.). Muthén & Muthén. https://www.statmodel.com

[CR33] Muthén, L. K., & Muthén, B. O. (2013). Version 7.1 Mplus language addendum. *Los Angeles, CA: Author*.

[CR34] Muthén BO (1984). A general structural equation model with dichotomous, ordered categorical, and continuous latent variable indicators. Psychometrika.

[CR35] Pendergast LL, von der Embse N, Kilgus SP, Eklund KR (2017). Measurement equivalence: A non-technical primer on categorical multi-group confirmatory factor analysis in school psychology. Journal of School Psychology.

[CR36] Penfield RD, Lam TCM (2005). Assessing differential item functioning in performance assessment: Review and recommendations. Educational Measurement: Issues and Practice.

[CR37] Pohl S, Schulze D, Stets E (2021). Partial measurement invariance: Extending and evaluating the cluster approach for identifying anchor items. Applied Psychological Measurement.

[CR38] Putnick DL, Bornstein MH (2016). Measurement invariance conventions and reporting: The state of the art and future directions for psychological research. Developmental Review.

[CR39] R Core Team. (2022). R: A language and environment for statistical computing [Manual]. R Foundation for Statistical Computing. https://www.R-project.org/

[CR40] Radloff LS (1977). The CES-D scale: A self-report depression scale for research in the general population. Applied Psychological Measurement.

[CR41] Revelle, W. (2022). Psych: Procedures for psychological, psychometric, and personality research [Manual]. Northwestern University. https://CRAN.R-project.org/package=psych

[CR42] Rhemtulla M, Brosseau-Liard PÉ, Savalei V (2012). When can categorical variables be treated as continuous? A comparison of robust continuous and categorical SEM estimation methods under suboptimal conditions. Psychological Methods.

[CR43] Rosseel, Y. (2012). Lavaan: An R package for structural equation modeling. *Journal of Statistical Software*, *48*(2), 1–36. 10.18637/jss.v048.i02

[CR44] Sass DA, Schmitt TA, Marsh HW (2014). Evaluating model fit with ordered categorical data within a measurement invariance framework: A comparison of estimators. Structural Equation Modeling: A Multidisciplinary Journal.

[CR45] Satorra A, Bentler PM (2001). A scaled difference chi-square test statistic for moment structure analysis. Psychometrika.

[CR46] Schmitt N, Kuljanin G (2008). Measurement invariance: Review of practice and implications. Human Resource Management Review.

[CR47] Sharman LS, Dingle GA, Vanman EJ (2019). Does crying help? Development of the beliefs about crying scale (BACS). Cognition and Emotion.

[CR48] Svetina, D., Rutkowski, L., & Rutkowski, D. (2019). Multiple-group invariance with categorical outcomes using updated guidelines: An illustration using M plus and the lavaan/semtools packages. *Structural Equation Modeling: A Multidisciplinary Journal,**27*(1), 111–130. 10.1080/10705511.2019.1602776

[CR49] Tay L, Meade AW, Cao M (2015). An overview and practical guide to irt measurement equivalence analysis. Organizational Research Methods.

[CR50] Teresi JA (2006). Overview of quantitative measurement methods: Equivalence, invariance, and differential item functioning in health applications. Medical Care.

[CR51] Thurstone, L. L. (1947). *Multiple-factor analysis: A development and expansion of the vectors of mind*. University of Chicago Press.

[CR52] Vandenberg RJ (2002). Toward a further understanding of and improvement in measurement invariance methods and procedures. Organizational Research Methods.

[CR53] Velicer WF (1976). Determining the number of components from the matrix of partial correlations. Psychometrika.

[CR54] Widaman, K. F., & Reise, S. P. (1997). Exploring the measurement invariance of psychological instruments: Applications in the substance use domain. In K. J. Bryant, M. Windle, & S. G. West (Eds.), *The science of prevention: Methodological advances from alcohol and substance abuse research.* (pp. 281–324). American Psychological Association. 10.1037/10222-009

[CR55] Widaman KF, Revelle W (2022). Thinking thrice about sum scores, and then some more about measurement and analysis. Behavior Research Methods.

[CR56] Wirth, R. J., & Edwards, M. C. (2007). Item factor analysis: Current approaches and future directions. *Psychological Methods,**12*(1), 58–79. 10.1037/1082-989X.12.1.58 10.1037/1082-989X.12.1.58 PMC3162326 17402812

[CR57] Wu H, Estabrook R (2016). Identification of confirmatory factor analysis models of different levels of invariance for ordered categorical outcomes. Psychometrika.

[CR58] Yoon, M., & Kim, E. S. (2014). A comparison of sequential and nonsequential specification searches in testing factorial invariance. *Behavior Research Methods,**46*(4), 1199–1206. 10.3758/s13428-013-0430-2 10.3758/s13428-013-0430-2 24356995

[CR59] Yoon M, Lai MHC (2018). Testing factorial invariance with unbalanced samples. Structural Equation Modeling: A Multidisciplinary Journal.

[CR60] Yoon, M., & Millsap, R. E. (2007). Detecting violations of factorial invariance using data-based specification searches: A monte carlo study. *Structural Equation Modeling: A Multidisciplinary Journal,**14*(3), 435–463. 10.1080/10705510701301677

